# The effect of systemic antidepressant treatments in early stage on neurocognitive function of euthymic bipolar patients initiated with a depressive onset: An observational, cross‐sectional, single‐blind study protocol

**DOI:** 10.1002/brb3.2360

**Published:** 2021-09-14

**Authors:** Huizeng Yang, Yuanyuan Liu, Chenghao Yang, Xiaoling Lin

**Affiliations:** ^1^ Tianjin Mental Health Center Tianjin Anding Hospital Tianjin China; ^2^ School of Nursing Sun Yat‐sen University Guangzhou China

**Keywords:** antidepressant treatment, bipolar disorder, euthymic state, neurocognitive function

## Abstract

**Objectives:**

Patients with bipolar disorder (BD) have a wide range of neurocognitive dysfunction even in euthymic state, leading to impaired psychosocial function and reduced quality of life. However, the understanding on risk factors related to neurocognitive impairment in such group of people is limited. In view of significantly delayed diagnosis of BD and common use of antidepressants prior to the BD diagnosis, the study aims to clarify whether systemic antidepressant use in early stage, defined as from the initial depressive episode to the diagnosis of BD, could impact neurocognitive function of euthymic bipolar patients.

**Methods:**

It is an observational, cross‐sectional, single‐blind trial, making a comparison in neurocognitive function between euthymic bipolar patients who had a depressive episode as initial onset and being with and without systemic antidepressant treatments in early stage (*n* = 62 and 62, respectively); secondary outcomes include the impact of systemic antidepressant use on global function, quality of life, sleep quality, positive and negative affect, and peripheral level of neuron‐specific enolase.

**Discussion:**

The study will provide a comprehensive and in‐depth understanding on the effect of systemic antidepressant treatments in early stage in such group of patients. It is expected to better guide the related prevention and treatment work of BD management.

**Trial Registration:**

The study was registered on Clinicaltrials.gov with protocol ID (TJAH2020‐18) and clinicaltrials.gov ID (NCT04564573).

## Graphical abstract

The study will provide a comprehensive and in‐depth understanding on the effect of systemic antidepressant treatments in early stage in bipolar patients initiated with a depressive episode. It is expected to better guide the related prevention and treatment work of bipolar disorder management.

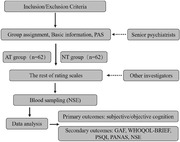

## 1 BACKGROUND

Bipolar disorder (BD) is a severe chronic disorder characterized by depressive episodes and alternating or intertwining manic and hypomanic episodes, with a lifetime prevalence rate exceeding 1% worldwide (Clemente et al., 2015). BD can result in a wide range of neurocognitive dysfunctions, mainly involving attention, verbal learning, nonverbal memory, and executive functions (Douglas et al., 2018; Kurtz & Gerraty, 2009). The neurocognitive dysfunctions could occur in very early stage of illness and persist even in euthymic period (Sole et al., 2017), which causes severe damage to individual psychosocial function and significantly increases disease‐related burden (Alonso et al., 2011). Therefore, alleviating neurocognitive dysfunction and accordingly improving patients’ psychosocial function and quality of life have become a critical goal of BD management (Grande et al., 2017). To this end, some clinical studies have attempted to improve the neurocognitive function of patients with BD pharmacologically and psychotherapeutically, but the outcomes are far from satisfaction (Sole et al., 2017). One of the reasons could be that the understanding on the risk factors related to neurocognitive impairment in such group of people is limited. Theoretically, a comprehensive and in‐depth understanding of relevant risk factors will help to better guide the prevention and treatment work of BD.

About 50% of BD initiates its expression in the form of depressive episode (Drancourt et al., 2013; Tondo et al., 2010), in which only 20% can get a diagnosis of BD within the first year of onset (Hirschfeld et al., 2003), and the average delay from first episode to diagnosis of BD is 5–10 years (Baldessarini et al., 2007). The main reason for this significant delay is that a large number of bipolar patients do not experience any manic/hypomanic symptoms in the early stage, defined as a term from initial depressive onset to the diagnosis of BD, and the clinical manifestations of bipolar depression are highly similar to unipolar depressive episodes (Drancourt et al., 2013; Tondo et al., 2010). As a result, patients may receive systemic treatment with antidepressants for a long time because of the misdiagnosis or delayed diagnosis. The attitude regarding the effect of antidepressants used in early stage on neurocognitive function of patients with BD is controversial yet. Empirically, antidepressant treatment could deprave the natural course of disease and facilitate switching from depressive onset to episodes of mania, hypomania, or mixed state (Berkol et al., 2019; Sole et al., 2017; Viktorin et al., 2014), which may cause cognitive injury (Goodwin et al., 2008). For example, BD patients with higher number of manic and/or hypomanic episodes had a worse performance in Neurocognitive Composite Index (NCI), visual memory, and working memory (Sánchez‐Morla et al., 2019); furthermore, patients with BD, particularly those with multiple mood episodes, showed severe dysfunction in emotional memory than healthy controls (Fijtman et al., 2020). In addition, the number of manic episodes was positively correlated with level of 8‐hydroxy‐2 deoxyguanosine (8‐OHdG), a modulator of DNA methylation, which could be a potential mechanism underlying cognitive dysfunction (Soeiro‐de‐Souza et al., 2013). Consistently, studies have shown that antidepressant agents per se undermined the neurocognitive function of the elderly and increased the incidence of mild cognitive impairment or dementia (Leng et al., 2018; Moraros et al., 2017), which may contribute partly to the neurocognitive impairments in patients with BD. However, it is arguable to expand the view based on the evidence from bipolar depression to depressive episode prior to any mania/hypomania emergence. Furthermore, it has been illustrated that antidepressant treatment can improve cognitive performance (Brendel et al., 2018; Tunc‐Ozcan et al., 2019), although the evidence did not derive from studies focusing on bipolar patients specifically. In view of the high incidence of delayed diagnosis or misdiagnosis of BD (Tondo et al., 2010), the use of antidepressants in the early stage is quite common and extensive. Therefore, a further understanding in the relationship between antidepressant used in the early stage and neurocognitive function of bipolar patients has significantly scientific and clinical significance.

The current study attempts to clarify this question by making comparisons in neurocognitive function between bipolar patients who had a depressive episode as initial onset and being with and without systemic antidepressant treatment (received adequate dosage of antidepressants for 6 weeks at least) in early stage. Furthermore, the social functions and quality of life will also be investigated because both items are highly associated with cognitive functions and are indicative for functional rehabilitation. Apart from those, we will also study the influence of antidepressants used in early stage on impulsiveness, quality of sleep, and positive and negative affect. Lastly, peripheral neuron‐specific enolase (NSE) will be evaluated. NSE, a cell‐specific isoenzyme of glycolytic enolase, is known as a sensitive marker of neuronal damage (Isgrò et al., 2015). It has been showed that high level of serum NSE was related to mild cognitive dysfunction in patients with diabetic retinopathy (Yu et al., 2020) and that serum NSE levels of BD patients were significantly different from healthy controls (Akcan et al., 2018). In this regard, it is expected to examine the potential association between serum NSE level and cognitive function of BD patients. Finally, although it is mainly an explorative investigation and is informative for clinical practice regardless of outcomes, we expect that systemic antidepressant treatments in early stage could negatively affect the neurocognitive function, social functions, and quality of life of patients with BD.

## 2 METHODS/DESIGN

### 2.1 Study design

This is a cross‐sectional study focusing on euthymic bipolar patients who had a depressive episode as initial onset. Participants were divided into two groups according to whether they had received systemic antidepressant treatment in the early stage: systemic antidepressant treatment (AT) group versus no systemic antidepressant treatment (NT) group.

### 2.2 Setting

All procedures will be performed in Tianjin Anding Hospital.

### 2.3 Population

This study will consist of a sample of 124 patients with BD (AT group vs. NT group: 62 vs. 62) recruited from outpatient and inpatient departments of Tianjin Anding Hospital.

### 2.4 Inclusion criteria

Patient's inclusion criteria are as follows: (1) diagnosisof BD according to the Diagnostic and Statistical Manual of Mental Disorders, Fourth Edition (DSM‐IV‐TR) with Structured Clinical Interview for DSM‐IV (SCID); current state of euthymia is defined by both scores of Montgomery–Åsberg Depression Scale (MADRS) and Young Manic Rating Scale (YMRS) < 7 (Cullen et al., 2016), lasting for 4 weeks at least before recruitment; (2) initiated with a depressive episode diagnosed with the MINI‐International Neuropsychiatric Interview (MINI); (3) age between 18 and 50 years; (4) participants comply with all procedures of study; and (5) participants must sign informed consent.

### 2.5 Exclusion criteria

Patient's exclusion criteria are as follows: (1) history of psychotic symptoms; (2) comorbidity of attention deficit and hyperactivity disorder; (3) neurological trauma or neurological diseases that could cause cognition injury; (4) history of substance dependence/abuse; (5) received modified electroconvulsive therapy in the past 12 months; (6) severe physical disease affecting cognitive function or increasing peripheral NSE; (7) recent drug use that affects cognition, such as tricyclic antidepressants, anticholinergic drugs, amphetamines, and so forth, (8) IQ < 70; and (9) use of benzodiazepines 4 h before scale evaluation.

### 2.6 Sample size calculation

There are no data available on effect size and standard deviations specific for cognitive function of targeted patients, therefore power calculation remains speculative. For the value of *δ*, we assume that it should come out between 0.25 and 0.5, and we set it as 0.3. With formula *n*_1_ = *n*_2_ = 2 × [(*t*_α/2_ + *t*_β/2_) *S*/*δ*]^2^, setting *α* at 0.05 and *β* at 0.20 in a two‐tailed independent *t*‐test indicated that a minimum of 62 patients per group is needed (totally 124 participants).

### 2.7 Study endpoint

Main study endpoints include neurocognitive functioning, which can be assessed by:

1. Subjective cognitive measures: The Cognitive Complaints in Bipolar Disorder Rating Assessment (COBRA) (Rosa et al., 2013).
2. Objective cognitive measures: The Stroop Color and Word Test (SCWT) (Golden, 1978), the categorical verbal fluency test (CVFT) (animal naming) (Chen et al., 2000), Chinese Auditory Verbal Learning Test (C‐AVLT) (Lee et al., 2002), and the Trail Making Test Part B (TMT‐B) (Lu & Bigler, 2000); the Digital Span Forward and Backward subtest (DSFB) and the Digit Symbol Coding subtest (DSC) of the Wechsler Adult Intelligence Scale‐Revised by China (WAIS‐RC) (Gong, 1982) and Trail Making Test Part A (TMT‐A) (Lezak et al., 2004); and the Visual Reproduction subtest (VRP) and the Visual Recognition subtest (VRC) of the Wechsler Memory Scale‐Revised (WMS‐R) (Gong, 1987).

Secondary study endpoints are as follows: Global Assessment Function (GAF), Methods Checklist on Quality of Life issued by World Health Organization‐Brief version (WHOQOL‐BRIEF), Pittsburgh Sleep Quality Index (PSQI), Positive and Negative Affect Scale (PANAS), and neuron‐specific enolase (NSE).

### 2.8 Duration

Information collection and scale rating for each patient will be finished within 180 min, and the whole enrollment work is expected to be finished in 12 months.

### 2.9 Additional study parameters

The information on age, sex, nation, family history of psychiatric disorder, age of onset, time of BD diagnosis, education level, use of mood stabilizer in early stage, number of depressive and manic episodes, history of euthymia (yes/no), and premorbid adjustment ability will be collected.

### 2.10 Blinding and study group allocation

Subjects will be divided into AT or NT group according to whether they had a history of receiving systemic antidepressant treatment in early stage. The screening process will be finished by two senior psychiatrists, and then the following rating works will be handed over to other investigators, which could keep the latter investigators blind to the subjects’ group assignments.

### 2.11 Study procedures

**FIGURE 1:**
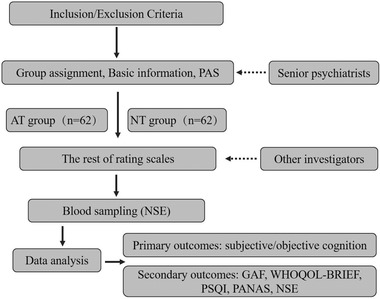
Study flowchart AT, systemic antidepressant treatment; GAF, Global Assessment Function; NSE, neuron‐specific enolase; NT, no systemic antidepressant treatment; PANAS, Positive and Negative Affect Scale; PAS, premorbid adjustment scale; PSQI, Pittsburgh Sleep Quality Index; WHOQOL‐BRIEF, Methods Checklist on Quality of Life issued by World Health Organization‐Brief version.

Subjects will not be engaged with any interventions in this study. Two senior psychiatrists are in attendance of screening the patients along the inclusion/exclusion criteria, and then all patients who meet both criteria and are willing to participate in this study will sign the informed consent before enrollment. Especially, all patients will be screened with Hypomania Check List‐32 items (HCL‐32) to detect the potential hypomanic symptoms in early stage. The participants will be divided into AT or NT group according to their history of systemic antidepressant treatment in early stage, which is done by the two senior psychiatrists. Then the senior psychiatrists will also collect additional study parameters, including age, sex, nation, age of onset, and score of Premorbid Adjustment Scale (PAS). Furthermore, the information about history of treatments and diagnosis will be further confirmed with medical documentary and patients’ guardians. Afterward, other investigators will finish the rating work of the remaining scales. The whole procedure should be done within 180 min. Finally, 10 ml of venous blood is adopted for measuring NSE (see Figure 1).

### 2.12 Scale assessments

COBRA will be used to assess subjective cognitive function, including verbal learning and memory, working memory, processing speed, executive functions, attention/concentration, and mental tracking. The summed scores of each item yield a total COBRA score, which is negatively correlated with subjective cognitive impairments.

SCWT, CVFT, and TMT‐B will be used to assess executive function, with higher scores indicating worse cognitive functioning.

DSFB, DSC of WAIS‐RC, and TMT‐A will be used to assess attention and processing speed. Higher scores for TMT‐A represent worse cognitive performance, and higher scores for DSFB and DSC of WAIS‐RS indicate better cognitive functioning.

VRP and VRC of WMS‐R will be used to assess visual memory, with higher scores indicating better cognitive functioning.

C‐AVLT will be used to measure verbal learning and memory domain. The higher number of word recall is associated with better verbal memory.

WHOQOL‐BRIEF will be used to assess the quality of life for the last 2 weeks prior to the assessment. It measures perceived quality of life for the domains of psychological health, physical health, social relationships, and environment as well as satisfaction with general health.

PSQI will be used to evaluate the quality of sleep for the last month, with a summed score ranging from 0 to 21, and a higher score indicates a worse sleeping quality.

PANAS will be used to detect the change in positive and negative affect. It is a self‐report instrument measuring the affective component of subjective well‐being, in which a higher score for each item refers to a higher degree for positive or negative affect.

MADRS will be used to assess the severity of depressive symptoms. The scale score ranges from 0 to 60, with a cutoff of 12 for depressive state.

YMRS will be used to assess the severity of manic symptoms. It includes 11 items, with a score not less than 6 indicating a manic state.

PAS will be used to evaluate the premorbid adjustment ability (Shi et al., 2013). PAS was designed for quantifying premorbid status in patients with schizophrenia originally, and now it has also been used in other field such as the study of mood disorders, coming to be regarded as a gold standard of retrospective assessment instrument.

MINI will be used to diagnose the initial episode of depressive disorder retrospectively.

HCL‐32 will be used to detect the potential hypomanic symptoms in early stage. It contains 32 items with “yes” and “no” options. When 14 items out of 32 items are “yes,” it indicates a hypomanic or manic state.

GAF will be used to assess patients’ functions psychologically, socially, and professionally. It is rated on a scale of nine levels, with higher level referring to better functions.

### 2.13 Biomarker

Peripheral NSE will be used to test the potential injury of neuron. Ten milliliters of venous blood are adopted from each participant into an anticoagulant‐free vacuum tube. The blood will be immediately centrifuged at 4000 × *g* for 10 min, and serum is kept frozen at −80°C until analysis. NSE levels are measured with electrochemiluminescence assay, using a commercial kit conforming to the manufacturer instructions (Roche Diagnostics, Mannheim, Germany) and data will be shown in ng/ml.

### 2.14 Data management

Data will be entered into OpenClinica online with range check. The academic board of Tianjin Anding Hospital will in charge of overseeing the trial including data monitoring, endpoint adjudication, and project implementation, which will be independent from investigators and the sponsor.

### 2.15 Medication use

None.

### 2.16 Side effects

None.

### 2.17 Safety reporting: Adverse events/serious adverse events

It is a cross‐sectional study without any interventions, and all assessments will occur during euthymic state. So, there is no need for AE/SAE monitoring based on the design of the study.

### 2.18 Withdrawal of subjects

Subjects can quit the study at any time for any considerations without any consequences. The investigator can decide to withdraw subjects from the trial if the subjects are not compliant with the study procedures or any conditions emerge that violate the inclusion/exclusion criteria during processing. All withdrawals will be excluded from the study and new participants will be recruited.

### 2.19 Statistical analysis

The data obtained from questionnaires and basic information are expressed in “mean ± standard deviation” in the case of normal distributions and in median and quartile in the case of nonnormal distributions. The comparisons in scores of each scale between two groups will be made by Covariance Analysis; the Chi Square and Analysis of Variance (ANOVA) test will be used to test the consistency of match between groups for factors such as age, sex, MADRS scores, YMRS scores, PAS scores, use of mood stabilizers, age of onset, number of attacks, and so forth, and the factors with significantly different distributions between two groups will be used as covariates. Furthermore, the two groups of patients will be combined as a whole and then Multiple Linear Regression equations will be used to analyze the linear relationship between each factor and neurocognitive function. The test level is α = 0.05.

## 3 DISCUSSION

Regarding the critical role of cognitive impairment in psychosocial disability and poorer quality of life, improving cognitive function has become a crucial goal in the management of BD patients. In this regard, increasing amount of research examining pharmacological and psychotherapeutic treatments was performed during the past decade (Alda et al., 2017; Solé et al., 2015). The research outcomes are inconsistent and no therapeutic intervention with sufficient efficacy is well‐established for improving cognitive dysfunction of BD yet (Miskowiak et al., 2016), although some of psychotherapeutic treatments or drugs appear as promising candidates. Many factors contributed to the discrepant outcomes such as poor randomization procedure, age of onset, number of depressive and manic episodes, severity of symptoms, and medications used specifically. In view of significantly delayed diagnosis of BD and common use of antidepressants in early stage, the effect of antidepressant agents on cognitive function has attracted increasing attention. But the data are still relatively rare, and the evidence is controversial. For example, a meta‐analysis estimated the relationship between antidepressant use and cognitive impairment and found that the odds of cognitive impairment were positively associated with antidepressant use in participants with an average age equal to or more than 65 years (odds ratio [OR] = 1.65), whereas those with participants aged less than 65 revealed an even stronger association (OR = 3.25) (Moraros et al., 2017); and also the study by Leng and colleagues illustrated that users of selective serotonin reuptake inhibitors and trazodone were as more as two to three times to develop dementia compared with the nonusers 5 years later, although the use of tricyclic antidepressants or other antidepressants was not involved in cognitive impairment (Leng et al., 2018). The evidence above suggests that antidepressant use could be a contributor for cognitive impairment of patients with mood disorder. Importantly, it is believed empirically that antidepressant treatment could deprave the natural course of BD and switch from depressive episode to episode of mania, hypomania, or mixed state (Viktorin et al., 2014), which may undermine the cognitive performance of bipolar patients (Goodwin et al., 2008). For instance, it was found that the higher number of manic and/or hypomanic episodes was negatively correlated with the cognitive performance of euthymic BD patients (*n* = 99) such as NCI, visual memory, and working memory in a 5‐year follow‐up longitudinal study (Sánchez‐Morla et al., 2019); furthermore, BD patients (*n* = 33), especially those with multiple mood episodes including mania, hypomania, and/or mixed state, showed severe impairments in emotional memory than healthy controls (*n* = 20) (Fijtman et al., 2020). Considering the significantly delayed diagnosis of BD and common use of antidepressants in clinical practice, such potential scathing effects should gather more attention. In contrast, another meta‐analysis suggested that antidepressants played a significant positive role in promoting psychomotor speed and delayed recall, with no significant difference between diverse types of antidepressants (Rosenblat et al., 2015). This is in line with the evidence from preclinical research that antidepressant agents could promote executive function and environmental information processing through integrating newborn neural cells into the hippocampal dentate gyrus (Tunc‐Ozcan et al., 2019). However, these data rarely came from clinical trial specifically focusing on the use of antidepressants in bipolar patients in early stage. To this end, the current study aims to clarify this issue by examining the cognitive function between bipolar patients who initiated with depressive episode and being with and without systemic antidepressant treatments in early stage. Additionally, the contemporary management of disease not only requires symptomatic relief but also pursues functional rehabilitation. Therefore, we will also examine the effect of antidepressant treatments in early stage on global function and quality of life of participants because both aspects are highly associated with cognitive function. Considering the potential influence of antidepressant use on progressing of course, the information on sleep quality and positive and negative affect will also be collected. Finally, the peripheral level of NSE will be tested to explore the potential biological mechanism of cognitive impairment.

To increase the power of this study, we have some considerations as following. First, we will perform the PAS measurement to adjust the potential difference in premorbid function that may confound the cognitive performance of participants. Second, it is required that participants should have a euthymic state for 4 weeks at least before enrollment because the duration in euthymia is associated with the quality of life (Pascual‐Sánchez et al., 2019), which has been found associated to cognitive function (Sánchez‐Morla et al., 2009). Third, we have two separate groups of investigators before and after group assignment to keep the later investigators blind to the assignment and then to avoid subjective bias on assessment of scales. Lastly, we set the range of age between 18 and 50 years to decrease the influence of aging in cognitive performance.

### 3.1 Limitations

There are some limitations of the current study. First, some information, such as history of antidepressant treatment and first manic/hypomanic episode, is mainly derived from patients’ memory, which may lead to a recall bias. For another, we assume a systemic antidepressant treatment as a consecutive therapy with adequate dosage of antidepressants for 6 weeks at least, but regardless of the antidepressant types and dose differences, which may obscure the potential effect of specific type or dose of drug. Finally, there are no data available on standard deviations and effect size for neurocognitive function of targeted patients, therefore the putative sample size might increase the risk of type II error.

## 4 CONCLUSION

In summary, despite these limitations, the present study is expected to help us look further into the effect of antidepressant use in early stage on the cognitive function of patient with BD and may provide clues for inspecting the pathophysiological association/discrepancy between unipolar depression and bipolar depression.

### PEER REVIEW

The peer review history for this article is available at https://publons.com/publon/10.1002/brb3.2360.

## CONFLICT OF INTEREST

The authors declare no conflict of interest.

## AUTHOR CONTRIBUTIONS

HY and CY conceived the study. YL and HY drafted the first version of manuscript. YL and XL provided the methodological supports. CY acquired funding. CY and XL finished the review and editing of protocol. All authors provided input and read and approved the final version.

## Data Availability

The related data and materials are available from the corresponding author on reasonable request.
